# Effects of omega-3 fatty acid supplementation on nutritional status and inflammatory response in patients with stage II-III NSCLC undergoing postoperative chemotherapy: a double-blind randomized controlled trial

**DOI:** 10.3389/fnut.2023.1266584

**Published:** 2023-10-26

**Authors:** Long Gui, Mingjin Cheng, Min Zheng, Chengdong Ning, Qianlun Huo

**Affiliations:** ^1^Department of Cardiothoracic Surgery, Lu’an Hospital Affiliated to Anhui Medical University, Lu’an, China; ^2^Department of Cardiothoracic Surgery, Lu’an People’s Hospital, Lu’an, China; ^3^Department of Nursing, Lu’an Hospital Affiliated to Anhui Medical University, Lu’an, China

**Keywords:** lung cancer, chemotherapy, inflammatory response, nutritional status, omega-3 fatty acids

## Abstract

**Background:**

The primary objective of this study was to investigate the effects of oral omega-3 fatty acids in lowering the risk of malnutrition and improving the inflammatory response in patients with stage II-III lung cancer receiving postoperative chemotherapy.

**Methods:**

One hundred and three lung cancer patients identified as being at risk for malnutrition according to the 2002 nutritional risk screening criteria were randomized into either the omega-3 fatty acid supplementation group or the placebo group during postoperative chemotherapy. Data on anthropometric parameters, laboratory nutritional indicators, and inflammatory markers were collected, and changes and differences between the two groups were compared and analyzed.

**Results:**

Sixty three patients were included in the final analysis. The baseline information of the two groups of patients was comparable (*p* > 0.05). After 12 weeks, patients in the treatment group exhibited significantly higher levels of hemoglobin (11.26 ± 1.25 vs.10.60 ± 0.94, *p* = 0.021) and serum albumin (45.38 ± 5.06 vs.42.66 ± 5.06, *p* = 0.036) compared with those in the placebo group. Meanwhile, the levels of inflammatory factors C-reactive protein (2.16 ± 1.06 vs. 4.11 ± 1.72, *p* < 0.001), interleukin-1 (6.61 ± 2.19 vs.10.85 ± 3.61, *p* < 0.001), interleukin-6 (2.48 ± 1.20 vs. 4.53 ± 0.98, *p* < 0.001), interleukin-8 (9.26 ± 2.69 vs. 39.01 ± 6.53, *p* < 0.001), and tumor necrosis factor-α (1.88 ± 0.60 vs. 4.07 ± 0.97, *p* < 0.001) were significantly decreased in the treatment group. In contrast, differences in weight, BMI, upper arm circumference, triceps skinfold thickness, triglycerides, cholesterol, and IFN-γ between the two groups were not statistically significant (*p* > 0.05). Finally, in the treatment group, the levels of hemoglobin (10.89 ± 1.15 vs. 11.82 ± 1.21, *p* = 0.042), triglyceride (0.92 ± 0.29 vs. 1.03 ± 0.22, *p* = 0.043), and cholesterol (3.56 ± 0.82 vs. 4.23 ± 0.88, *p* = 0.045) were higher in stage II patients after the intervention compared with stage III patients.

**Conclusion:**

Supplementation with omega-3 fatty acids improved nutritional status and reduced chronic inflammatory responses in patients with stage II-III non-small cell lung cancer undergoing postoperative chemotherapy.

**Clinical Trial Registration:**

AEA RCT Registry, identifier AEARCTR-0007165.

## Introduction

As is well documented, lung cancer remains the leading cause of cancer-related deaths worldwide. According to GLOBOCAN statistics on cancer in 185 countries in 2020 (1), approximately 1.8 million individuals are estimated to die annually from lung cancer, accounting for 18% of the total cancer mortality. Its occurrence is closely related to smoking, air pollution, occupational exposure, and environmental factors (2–4). Surgical-based multimodal treatment is recommended for all patients eligible for surgery after a comprehensive evaluation. Meanwhile, postoperative adjuvant chemotherapy assists in eliminating postoperative residual cancer cells, thereby minimizing the risk of postoperative recurrence and improving patients’ postoperative survival time (5, 6).

However, postoperative chemotherapy is prone to cause adverse reactions such as nausea, vomiting, diarrhea, anorexia, neutropenia, and thrombocytopenia. Additionally, a proportion of patients manifest a deterioration in nutritional status, leading to decreased immune function, a higher risk of infection-related complications, and eventually cancer-related fatigue (7). These effects can have detrimental impacts on the physical, psychological, familial, and social well-being of patients. Consequently, enhancing the physical condition of postoperative patients has been the spotlight of the field of oncology.

Omega-3 polyunsaturated fatty acids (ω-3 PUFA), also referred to as n-3 PUFA, are fundamental components of a healthy human diet and encompass a-linolenic acid (ALA), eicosapentaenoic acid (EPA), docosahexaenoic acid (DHA), and docosapentaenoic acid (DPA) (8). ALA is an essential fatty acid found mainly in plant oils. DPA is a vital intermediate product aiding in the conversion of ALA into EPA and DHA, yet the human body is unable to efficiently synthesize it owing to the low activity of enzymes involved in its conversion, resulting in a drastic limitation of its conversion capacity (9). Earlier studies have reported that ω-3 PUFA supplementation can increase skeletal muscle mass, modulate inflammatory responses, reduce the risk of gastrointestinal reactions, attenuate anorexia, improve the prognosis of cancer patients, and confer tolerance to chemotherapy, radiotherapy, and surgery in patients with non-small cell lung cancer (NSCLC), thus prolonging their survival time (10–14).

Numerous studies have extensively studied and established the anti-inflammatory and immunomodulatory benefits of ω-3 PUFA in cancer patients (15–18). Nevertheless, studies on its effect on lung cancer patients at risk of malnutrition receiving postoperative chemotherapy are scarce. Furthermore, clinical studies have reported that the influence of ω-3 PUFA on cancer progression and nutritional status is controversial (19). Therefore, this study aimed to investigate the effect of ω-3 PUFA on the nutritional status and inflammatory response of postoperative NSCLC patients receiving chemotherapy.

## Materials and methods

### Study design

This was a double-blind, randomized, placebo-controlled trial. One hundred and three patients who attended the Department of Thoracic Surgery of Lu’an Hospital, Anhui Medical University, from May 2021 to December 2022 and were diagnosed with NSCLC by postoperative pathological biopsies were recruited. Nutritional risk Screening 2002 (NRS-2002) was used to assess the clinical nutritional risk of each patient before surgery, and this scoring scale was used to identify the malnutrition of patients. These patients were randomized to either the ω-3 PUFA supplementation group or the placebo group. This study was approved by the Ethics Committee of the Affiliated Lu’an Hospital of Anhui Medical University (approval number: 2021LL009) and conformed to the Declaration of Helsinki, and all patients were fully informed and signed the informed consent form.

### Inclusion criteria

Patients preoperatively assessed for surgical intervention, with an NRS-2002 score equal to or greater than 3 points (risk of malnutrition), histopathology-confirmed postoperative stage II-III NSCLC, postoperative assessment of life expectancy exceeding 3 months, and consent to continue with postoperative adjuvant chemotherapy.

### Exclusion criteria

Refusal to continue chemotherapy after surgery or intolerance to chemotherapy; intolerance to fish or fish oil preparations; poorly controlled cardiovascular and renal diseases, diabetes, gastrointestinal disease, and severe infections; refusal to participate in the randomized trial; incomplete information during the study.

### Intervention

Eligible patients were randomized by the clinical secretary according to a randomized list into two groups: The ω-3 PUFA supplementation group (treatment group) and placebo group. The treatment group received ω-3 polyunsaturated fatty acid gel capsules (1.6 g EPA/day and 0.8 g DHA/ day, no other additives or antioxidants), while the placebo capsules consisted of sunflower oil (2.4 g/day). The shape, size, and mass of the gel capsules were identical, and both investigators and patients were blinded to group assignment. The start of the intervention was 1 week postoperatively when there were no obvious complications, and they were instructed to take it before meals at a fixed time every day. A return form was distributed to record daily medication and adverse events and retrieved at each visit to the hospital for chemotherapy. Patients were encouraged to report perceived adverse events to the investigator by telephone or other means. The choice of chemotherapy regimen was based on the postoperative pathological type, chemotherapeutic drug sensitivity report and body surface area. All patients strictly adhered to the standard cancer treatment protocols.

### Assessment method

Anthropometric measurements reflect nutritional status, comprising weight, height, and body mass index (BMI). Patient body weight was measured under fasting conditions in the morning, without shoes, while wearing the same patient uniform. The right mid-arm circumference was also recorded. Triceps skinfold thickness was measured using a Harpenden caliper. Nutrition-related laboratory indicators were routinely tested by the clinical laboratory of the hospital. Among them, hemoglobin was determined by optical absorption, albumin was determined by turbidimetry, and triglycerides and cholesterol were determined by enzymatic methods. The levels of inflammatory markers [C-reactive protein (CRP), tumor necrosis factor-α (TNF-α), interleukin-1(IL-1), interleukin-6(IL-6), interleukin-8 (IL-8), and interferon-γ (IFN-γ)] were measured by mature ELISA kits. Specifically, CRP levels were determined using a Human High Sensitivity ELISA Kit (Anisan™, Tehran, Iran), whilst serum TNF-α, IL-1, IL-6, IL-8, and IFN-γ levels were measured using a human cytokine-specific ELISA kit (Biosource Europe, Belgium). These kits have been widely used in multiple studies and experiments, and their performance and stability have been thoroughly validated.

### Statistical analysis

Statistical analyses were conducted using SPSS version 22.0 for Windows (SPSS Inc., Chicago, IL, United States). The Shapiro–Wilk method was used to assess data normality. Measurement data were expressed as mean ± standard deviation (Mean ± SD), and independent samples *t*-test was used to compare the means of two consecutive normally distributed variables. The Mann–Whitney U test was employed to compare the means of two groups of non-normally distributed variables. The Pearson χ^2^-test was used to compare the proportions of two variables. The hypothesis test level was set to *α* = 0.05 and was considered statistically significant at *p* < 0.05.

## Results

**Figure 1:**
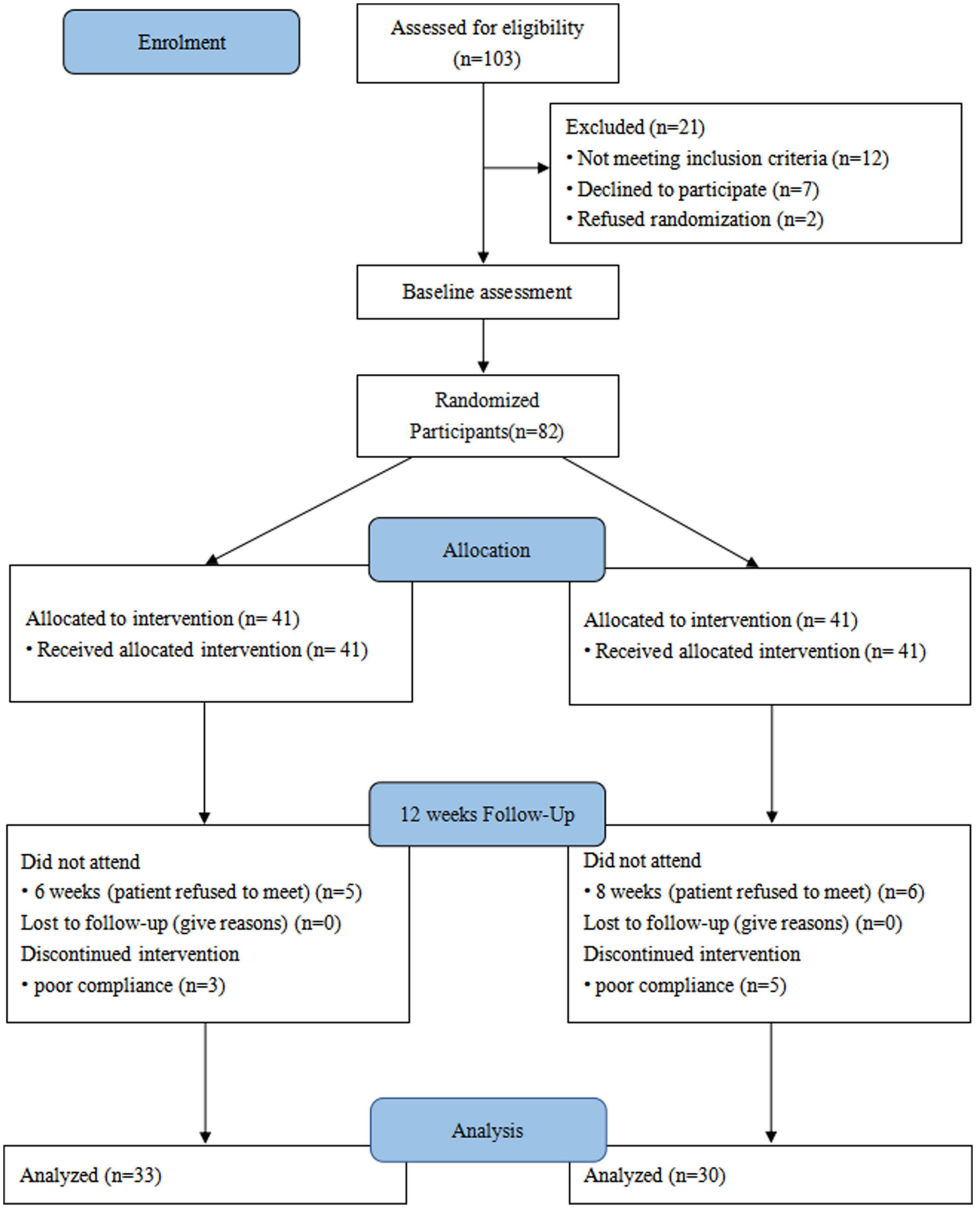
Consolidated standards of reporting trials (CONSORT) diagram. A patient flow diagram is shown.

This trial initially enrolled 103 patients, of whom 12 did not meet the inclusion criteria for this trial, 7 declined to participate, and 2 requested direct inclusion in the treatment group and declined random assignment based on clinical data and eligibility criteria. Based on a computerized random assignment list, 82 patients were randomly assigned to the treatment and placebo groups. After 12 weeks of intervention, patient follow-up results were recorded. In the treatment group, 5 patients were excluded due to poor adherence, and 6 additional patients who refused to participate in follow-up visits were also excluded. In the placebo group, five patients refused to participate in follow-up visits, and three were excluded owing to poor adherence to medications. Finally, 63 subjects were enrolled (Figure 1). The treatment group (*n* = 30) consisted of 18 males (60%) and 12 females (40%) with a mean age of (62.50 ± 4.88) years. Similarly, there were 20 males (60.6%) and 13 females (39.4%) in the placebo group (*n* = 33) with a mean age of (61.81 ± 7.15) years. The baseline demographics and clinical characteristics of enrolled participants were comparable (Table 1). Adverse reactions during chemotherapy in the two groups were found to be gastrointestinal symptoms (Treatment group, *n* = 18 vs. Placebo group, *n* = 21, *p* = 0.885), Blood system symptoms (Treatment group, *n* = 5 vs. Placebo group, *n* = 7, *p* = 0.646), other symptoms such as rash, insomnia, etc. (Treatment group, *n* = 2 vs. Placebo group, *n* = 3, *p* = 0.913). After 12 weeks of intervention, there were no statistical differences in anthropometric parameters between the two groups, including weight, BMI, upper arm circumference, and triceps skinfold thickness (Table 2). Among the nutrition-based laboratory indicators, hemoglobin (11.26 ± 1.25 vs. 10.60 ± 0.94), serum albumin (45.38 ± 5.06 vs. 42.66 ± 5.06), and the change of serum albumin before and after intervention (4.50 ± 6.34 vs. 0.32 ± 5.58) were significantly different between the two groups (*p* < 0.05). There was no significant difference in triglycerides and cholesterol levels after the intervention. However, there were significant differences in the changes of triglyceride and cholesterol before and after intervention between the two groups (*p* < 0.05).

**Table 1:** Baseline characteristics of participants.

Variable	Treatment group (*n* = 30)	Placebo group (*n* = 33)	*p*-value
Gender (N[%])			0.961
Male	18 (60.0)	20 (60.6)	
Female	12 (40.0)	13 (39.4)	
Age (year)	62.50 ± 4.88	61.81 ± 7.15	0.658
NRS-2002	3.23 ± 0.50	3.24 ± 0.50	0.943
Type of NSCLC (N[%])			
Adenocarcinomas	16 (53.3)	19 (57.6)	0.735
Squamous-cell carcinoma	11 (36.7)	10 (30.3)	0.593
Large cell carcinomas	1 (3.3)	1 (3.0)	0.945
Others	2 (6.7)	3 (9.1)	0.722
Stage of NSCLC (N[%])			
IIa	9 (30.0)	12 (36.4)	0.533
IIb	9 (30.0)	7 (21.2)	0.424
IIIa	8 (26.7)	10 (30.3)	0.750
IIIb	4 (13.3)	3 (9.1)	0.593
IIIc	0 (0.0)	1 (3.0)	0.336
Chemotherapy regimens (N[%])			
Cisplatin	11 (36.7)	10 (30.3)	0.593
Cisplatin and docetaxel	15 (50.0)	17 (51.5)	0.904
Cisplatin and pemetrexed disodium	4 (13.3)	6 (18.2)	0.599

NRS, nutritional risk screening. All variables are expressed as mean ± standard deviation (Mean ± SD) or frequency (percent). The Pearson χ^2^-test was used for the comparison of gender, type of NSCLC, stage of NSCLC, and chemotherapy regimens, the Mann–Whitney U test was used for the comparison of NRS-2002 scores, and the independent samples *t-*test was used for the comparison of age.

**Table 2:** Nutritional status and blood biochemical indices of patients in treatment group and placebo group.

Characteristics		Treatment group (*n* = 30)	Placebo group (*n* = 33)	*p*-value
Weight (Kg)	Before	60.72 ± 5.41	60.51 ± 6.59	0.889*
	After	61.70 ± 5.40	61.29 ± 6.78	0.792*
	Δ	0.97 ± 1.73	0.78 ± 1.59	0.638*
Height (cm)		166.23 ± 4.76	165.73 ± 5.86	0.710*
BMI (Kg/m^2^)	Before	21.99 ± 1.97	22.02 ± 1.97	0.960*
	After	22.35 ± 2.04	22.29 ± 1.99	0.910*
	Δ	0.36 ± 0.63	0.27 ± 0.60	0.596*
Upper arm circumference (mm)	Before	24.24 ± 1.85	24.43 ± 1.62	0.670*
	After	24.41 ± 1.87	24.54 ± 1.58	0.767*
	Δ	0.17 ± 0.26	0.11 ± 0.40	0.688^#^
Triceps skinfold thickness (mm)	Before	12.31 ± 1.81	12.82 ± 2.02	0.296*
	After	12.51 ± 1.84	13.02 ± 1.98	0.295*
	Δ	0.20 ± 0.31	0.19 ± 0.26	0.591^#^
Hemoglobin (g/dL)	Before	10.64 ± 1.28	10.64 ± 1.42	0.994*
	After	11.26 ± 1.25	10.60 ± 0.94	0.021*
	Δ	0.62 ± 1.45	−0.04 ± 1.27	0.164^#^
Albumin (g/dL)	Before	40.89 ± 6.60	42.33 ± 6.00	0.336*
	After	45.38 ± 5.06	42.65 ± 5.06	0.036*
	Δ	4.50 ± 6.34	0.32 ± 5.58	0.006^#^
Triglyceride (mmol/L)	Before	0.95 ± 0.31	0.96 ± 0.24	0.308^#^
	After	0.96 ± 0.26	0.92 ± 0.25	0.685^#^
	Δ	0.01 ± 0.26	−0.04 ± 0.15	0.049^#^
Cholesterol (mmol/L)	Before	3.96 ± 0.92	3.84 ± 0.83	0.582^#^
	After	3.83 ± 0.89	3.95 ± 0.89	0.604*
	Δ	−0.13 ± 0.45	0.11 ± 0.42	0.030^#^

BMI, Body mass index; Δ, Difference between post-intervention and pre-intervention. All variables are expressed as mean ± standard deviation (Mean ± SD). ^*^p, Independent samples *t-*test for normally distributed continuous variables. ^#^*p*, Mann–Whitney U test for non-normally distributed continuous variables, *p* < 0.05 indicates a statistical difference.

Analyzing the results of inflammatory factor assays exposed that after the intervention, the levels of CRP and TNF-α were significantly lower in the treatment group compared with those in the placebo group (2.16 ± 1.06 vs. 4.11 ± 1.72, *p* < 0.001 and 1.88 ± 0.60 vs. 4.07 ± 0.97, *p* < 0.001). Likewise, the levels of IL-1, IL-6, and IL-8 were also significantly lower in the treatment group than those in the placebo group (6.61 ± 2.19 vs.10.85 ± 3.61, 2.48 ± 1.20 vs. 4.53 ± 0.98, and 9.26 ± 2.69 vs. 39.01 ± 6.53, respectively, *p* < 0.001). We found statistically significant differences between the two groups when comparing the differences in changes in CRP, IL-1, IL-6, IL-8, and TNF-α before and after the intervention (*p* < 0.05). Moreover, the level of IFN-γ was numerically lower in the treatment group than that in the placebo group (9.22 ± 2.86 vs. 10.50 ± 3.34), but the difference was not statistically significant (*p* = 0.099) (Table 3).

**Table 3:** Inflammatory factors of patients in treatment group and placebo group.

Variable		Treatment group (*n* = 30)	Placebo group (*n* = 33)	*p*-value
CRP (mg/L)	Before	4.89 ± 2.00	4.24 ± 1.36	0.208^#^
	After	2.16 ± 1.06	4.11 ± 1.72	<0.001*
	Δ	−2.73 ± 1.87	−0.13 ± 1.88	<0.001*
IL-1 (pg/mL)	Before	11.46 ± 4.81	11.15 ± 3.59	0.741^#^
	After	6.61 ± 2.19	10.85 ± 3.61	<0.001^#^
	Δ	−4.85 ± 5.10	−0.29 ± 4.52	<0.001*
IL-6 (pg/mL)	Before	4.71 ± 0.86	4.60 ± 0.98	0.831^#^
	After	2.48 ± 1.20	4.53 ± 0.98	<0.001*
	Δ	−2.23 ± 1.53	−0.07 ± 1.40	<0.001*
IL-8 (pg/mL)	Before	37.96 ± 6.69	38.72 ± 5.88	0.640^#^
	After	9.26 ± 2.69	39.01 ± 6.53	<0.001^#^
	Δ	−28.70 ± 8.12	0.28 ± 8.35	<0.001^#^
TNF-α (pg/mL)	Before	4.10 ± 1.17	3.97 ± 1.13	0.670*
	After	1.88 ± 0.60	4.07 ± 0.97	<0.001*
	Δ	−2.22 ± 1.19	0.09 ± 1.41	<0.001*
IFN-γ (pg/mL)	Before	10.44 ± 3.85	11.21 ± 2.99	0.376*
	After	9.22 ± 2.86	10.50 ± 3.34	0.099^#^
	Δ	−1.22 ± 4.68	−0.71 ± 4.02	0.645*

Δ, Difference between post-intervention and pre-intervention. All variables are expressed as mean ± standard deviation (Mean ± SD). *p, By independent samples *t*-test. ^#^*p*, By Mann–Whitney U test.

Comparing post-treatment laboratory parameters in patients with stage II (*n* = 18) and stage III (*n* = 12) NSCLC in the treatment group uncovered that the levels of hemoglobin (11.82 ± 1.21 vs. 10.89 ± 1.15, *p* = 0.042), triglycerides (1.03 ± 0.22 vs. 0.92 ± 0.29, *p* = 0.044) and cholesterol (4.23 ± 0.88 vs. 3.56 ± 0.82, *p* = 0.045) were significantly elevated in patients with stage III NSCLC compared to those with stage II NSCLC. The anthropometric indices and inflammatory factor levels were similar between stage II and III patients, and only the change in IFN-γ before and after the intervention was statistically different between the two stages (*p* < 0.05) (Table 4).

**Table 4:** Anthropometric parameters, nutritional indicators and inflammatory factors in patients with stage II and III NSCLC in the treatment group.

Variable		Stage II (*n* = 18)	Stage III (*n* = 12)	*p*-value
Weight (Kg)	Before	60.48 ± 6.31	61.09 ± 3.91	0.745*
	After	61.53 ± 6.36	61.95 ± 3.73	0.821*
	Δ	1.05 ± 1.71	0.86 ± 1.82	0.539^#^
BMI (Kg/m^2^)	Before	21.88 ± 2.34	21.16 ± 1.33	0.710*
	After	22.26 ± 2.34	22.49 ± 1.57	0.766*
	Δ	0.38 ± 0.62	0.33 ± 0.67	0.657^#^
Upper arm circumference (mm)	Before	24.30 ± 2.09	24.15 ± 1.51	0.832*
	After	24.46 ± 2.11	24.33 ± 1.54	0.849*
	Δ	0.16 ± 0.25	0.18 ± 0.29	0.797^#^
Skinfold thickness (mm)	Before	11.98 ± 1.55	12.82 ± 2.10	0.217*
	After	12.19 ± 1.64	12.99 ± 2.10	0.249*
	Δ	0.21 ± 0.33	0.17 ± 0.29	0.752*
Hemoglobin (g/dL)	Before	10.37 ± 1.32	11.04 ± 1.15	0.159*
	After	10.88 ± 1.15	11.82 ± 1.21	0.042*
	Δ	0.52 ± 1.52	0.78 ± 1.39	0.433^#^
Albumin (g/dL)	Before	40.41 ± 6.98	41.61 ± 6.21	0.525^#^
	After	45.28 ± 5.13	45.53 ± 5.17	0.897*
	Δ	4.88 ± 6.02	3.93 ± 7.04	0.694*
Triglyceride (mmol/L)	Before	0.95 ± 0.35	0.96 ± 0.25	0.341^#^
	After	0.92 ± 0.29	1.03 ± 0.22	0.044^#^
	Δ	−0.03 ± 0.31	0.07 ± 0.13	0.235^#^
Cholesterol (mmol/L)	Before	3.63 ± 0.89	4.44 ± 0.77	0.016*
	After	3.56 ± 0.82	4.23 ± 0.88	0.045*
	Δ	−0.07 ± 0.39	−0.21 ± 0.55	0.044^#^
CRP (mg/L)	Before	4.99 ± 2.11	4.74 ± 1.91	0.747*
	After	2.31 ± 1.03	1.95 ± 1.11	0.378*
	Δ	−2.68 ± 1.79	−2.79 ± 2.07	0.672^#^
IL-1 (pg/mL)	Before	12.51 ± 4.91	9.89 ± 4.39	0.176^#^
	After	6.89 ± 1.98	6.19 ± 2.49	0.374^#^
	Δ	−5.62 ± 5.70	−3.70 ± 4.00	0.322*
IL-6 (pg/mL)	Before	4.66 ± 0.87	4.78 ± 0.87	0.725*
	After	2.45 ± 1.38	2.52 ± 0.91	0.611^#^
	Δ	−2.22 ± 1.71	−2.26 ± 1.28	0.966^#^
IL-8 (pg/mL)	Before	38.11 ± 7.29	37.74 ± 5.97	0.966^#^
	After	9.24 ± 2.50	9.29 ± 3.07	0.799^#^
	Δ	−28.87 ± 8.91	−28.45 ± 7.14	0.866^#^
TNF-α (pg/mL)	Before	4.16 ± 1.11	4.01 ± 1.30	0.738*
	After	1.93 ± 0.60	1.80 ± 0.61	0.579*
	Δ	−2.23 ± 0.98	−2.20 ± 1.49	0.961*
IFN-γ (pg/mL)	Before	12.04 ± 3.69	8.05 ± 2.78	0.004*
	After	8.73 ± 2.74	9.96 ± 2.99	0.253*
	Δ	−3.31 ± 4.07	1.91 ± 3.77	0.001*

Δ, Difference between post-intervention and pre-intervention. All variables are expressed as mean ± standard deviation (Mean ± SD). *p, By independent samples *t*-test, ^#^*p*, By Mann–Whitney U test.

## Discussion

In the multimodal treatment of lung cancer, patients undergoing postoperative chemotherapy have garnered extensive attention. This randomized controlled trial investigated the influence of oral ω-3 PUFA in reducing the risk of malnutrition and the levels of inflammatory biomarkers in patients with stage II-III NSCLC undergoing postoperative adjuvant chemotherapy. Differences in metrics between the two randomly assigned groups suggested that supplementation of ω-3 PUFA during the early postoperative period lowered the risk of malnutrition in patients while alleviating the inflammatory response during chemotherapy.

Several studies have described that ω-3 PUFA results in beneficial health outcomes in various fields of medicine (20–23). Evidence from *in vitro* trials, studies utilizing animal experimental models, and epidemiological analyses advocate the use of ω-3 PUFA for multi-targeted tumor therapy, which has consistently yielded comparable results in clinical trials (24). Nonetheless, the mechanism by which ω-3 PUFA inhibits tumor progression has not been elucidated so far. ω-3 PUFA is hypothesized to regulate cell replication during proliferation and differentiation, interfere with cell cycle, and disrupt cell growth via the modulation of apoptosis and necrosis in tumor cells (25, 26). In addition, ω-3 PUFA can act through mechanisms such as inhibition of arachidonic acid derivatives, interference with oxygen radicals, and reactive oxygen species production (27, 28), all of which are based on the action of EPA and DHA. In a double-blind trial, Van der Meij et al. administered n-3 PUFA to non-surgically treated NSCLC patients while the other group received an isocaloric control supplement and evinced that EPA 2.02 + DHA 0.92 g positively enhanced not only physical activity but also the overall prognosis of NSCLC patients (29). Additional trials are warranted on the dose–response relationship of ω-3 PUFA, considering that the influence of its individual components on quality of life is elusive. According to previous studies, (19, 30–32) the administration of oral ω-3 PUFA (EPA1.6 g/day+ DHA 0.8 g/day) is safe and effective, in line with the findings of our previous study (33).

Patients undergoing post-operative chemotherapy frequently suffer from nausea, anorexia, anxiety, and even transient weight loss during the early stage. Indeed, any form of cancer treatment demands increased protein and energy support. Albumin is a good indicator to directly reflect the nutritional status of patients. The normal synthesis and maintenance of hemoglobin, a protein found in red blood cells, requires a variety of nutrients, including iron, vitamin B12, folate, protein, and vitamin C. Poor nutrition and an unbalanced diet can both lead to insufficient hemoglobin synthesis, which can cause anemia. Maintaining adequate nutrient intake is essential for maintaining healthy hemoglobin levels. In a study carried out by Kaya et al., tumor patients had significantly lower postoperative serum albumin levels compared to their preoperative levels, whereas patients on an ω-3 PUFA-enriched diet experienced a dramatic reduction in albumin loss, indicating that ω-3 PUFA contributed to the nutritional recovery of postoperative patients (34). This conclusion is in agreement with our current research results that ω-3 PUFA supplementation improves the nutritional status of postoperative patients. Specifically, significant differences in hemoglobin and serum albumin levels were observed in patients after 12 weeks of oral ω-3 PUFA supplementation compared to those in the placebo group. In contrast, despite increasing, anthropometric parameters of patients taking oral sunflower oil were comparable to baseline levels. This may be ascribed to differences in the included population and the duration of the study. Murphy et al. uncovered an interaction between plasma n-3 PUFA levels and the rate of skeletal muscle change in NSCLC patients during chemotherapy (2.5 months) sessions and pointed out that the accelerated rate of muscle loss in patients with concurrent sarcopenia was closely related to plasma n-3 PUFA deficiency (35). However, the majority of studies have used the change in values from clinical trials as the study endpoint and have provided elaboration or hypotheses on the mechanism.

Inflammation is an important marker of cancer, and many studies have identified chronic inflammation as correlated with a poor prognosis (36, 37). Cytokines such as IL-1, IL-6, IL-8, TNF-α, and IFN-γ play a role in promoting inflammation in the inflammatory response. When the body is exposed to infection, injury, or other inflammatory stimuli, these cytokines are released by immune cells, initiating the inflammatory process. They cause vasodilation, increase blood flow, and attract immune cells such as white blood cells to damaged areas to fight potential pathogens or repair tissue damage. A prospective randomized controlled study conducted by Liang et al. included 42 patients who underwent radical colorectal cancer surgery and received either soybean oil supplementation (SO group) or a combination of ω-3 PUFA and soybean oil (FO group) as total parenteral nutrition post-surgery and found that IL-6 and TNF-α levels were lower in patients in the FO group than those in the SO group during the early postoperative period (38). Weiss et al. also observed a significant decrease in IL-6 levels in perioperative patients following fish oil intervention, as well as a corresponding decrease in TNF-α released by monocytes (39). Consistent with our results, there was a significant decrease in the level of both IL-6 and TNF-α in patients taking ω-3 PUFA after a 12-week dosing intervention. Compared to the study by Weiss et al., our study focused on the effect of ω-3 PUFA in chemotherapy-induced chronic inflammation, given that acute inflammatory responses in the perioperative period may be influenced by confounding factors such as the duration of surgery, antibiotic use, and the resolution of complications.

CRP is regarded as a classical inflammatory biomarker in oncology studies (40, 41), and its level is correlated with malignant progression and treatment-associated complications in lung cancer patients (42, 43). TNF-α also participates in the development of malignancies as an endogenous tumor-promoting factor. A meta-analysis established that expression levels of inflammatory factors such as CRP, TNF-α, and IL-6 in adults administered n-3 PUFA were significantly low; consequently, n-3 PUFA could be considered and recommended as an adjuvant anti-inflammatory agent (44). In a clinical trial including 64 oncology patients, significant differences in TNF-α, IL-1β, IL-6, and IFN-γ levels were detected in patients supplemented with EPA 2.0 g per day, but ω-3 PUFA did not significantly lower CRP levels compared to the control group (45). This result differs from our results, wherein significant changes in the levels of inflammatory factors were identified in patients in the treatment group, with the exception of IFN-γ (*p* = 0.099). We hypothesize that this discrepancy may be attributed to the limited sample size in the current study and the varying levels of serum IFN-γ in patients with tumors originating from different sites.

In addition to this, in this investigation, we compared the corresponding changes in study outcomes in the treatment group for NSCLC before and after the administration of the drug for different periods. The level of hemoglobin and triglycerides did not differ in the treatment group before the intervention, while statistical differences appeared after 12 weeks of intervention, indicating that ω-3 PUFA was more effective than stage II patients in promoting a positive shift in nutritional risk in stage III NSCLC patients, while in terms of anti-inflammatory effects, ω-3 PUFA did not show a particular advantage for stage III patients. Because of the difference between the baseline values of cholesterol before intervention in the two stages of patients, although the prognosis has changed significantly, the role of cholesterol still needs further clinical research. Van der Meij also found that n-3 PUFA exerted an anti-inflammatory effect in patients with stage III NSCLC receiving multimodal therapy (29), although no within-group comparisons were made between stage IIIa and IIIb patients. Some limitations of our study should also be acknowledged. For instance, the possibility of sampling error and bias due to the small sample size of stage II and III patients cannot be excluded.

An innovative randomized controlled trial was conducted to prospectively explore the relationship between ω-3 PUFA, malnutrition, and inflammatory factors in a specific population. We postulate that ω-3 PUFA may serve as an adjuvant therapy for patients undergoing chemotherapy after lung cancer surgery. However, this study exclusively included patients at nutritional risk, ultimately resulting in a small sample size. Furthermore, the results may not be representative of a broader population, and the study possesses inherent limitations typical of trials of this nature. In addition, this study did not include a dose-escalation test of ω-3 PUFA and did not examine the dose–response effect on patients. Nonetheless, the results of this trial provide compelling evidence for the beneficial effects of ω-3 PUFA in NSCLC patients undergoing postoperative chemotherapy.

## Conclusion

ω-3 PUFA supplementation during postoperative adjuvant chemotherapy may enhance their nutritional status while mitigating inflammatory responses in patients at nutritional risk diagnosed with stage II-III NSCLC.

## Data availability statement

The original contributions presented in the study are included in the article/supplementary material, further inquiries can be directed to the corresponding author.

## Ethics statement

The studies involving humans were approved by the Ethics Committee of the Affiliated Lu’an Hospital of Anhui Medical University (approval number: 2021LL009). The studies were conducted in accordance with the local legislation and institutional requirements. The participants provided their written informed consent to participate in this study. Written informed consent was obtained from the individual(s) for the publication of any potentially identifiable images or data included in this article.

## Author contributions

LG: Investigation, Software, Writing – original draft. MC: Conceptualization, Supervision, Validation, Writing – review & editing. MZ: Data curation, Formal analysis, Writing – review & editing. CN: Methodology, Supervision, Writing – review & editing. QH: Project administration, Supervision, Writing – review & editing.

## References

[1] Sung H Ferlay J Siegel RL Laversanne M Soerjomataram I Jemal A. Global Cancer statistics 2020: GLOBOCAN estimates of incidence and mortality worldwide for 36 cancers in 185 countries. CA Cancer J Clin. (2021) 71:209–49. doi: 10.3322/caac.21660, PMID: 33538338

[2] Loomis D Guha N Hall AL Straif K. Identifying occupational carcinogens: an update from the IARC monographs. Occup Environ Med. (2018) 75:593–603. doi: 10.1136/oemed-2017-104944, PMID: 29769352 PMC6204931

[3] Raaschou-Nielsen O Andersen ZJ Beelen R Samoli E Stafoggia M Weinmayr G. Air pollution and lung cancer incidence in 17 European cohorts: prospective analyses from the European study of cohorts for air pollution effects (ESCAPE). Lancet Oncol. (2013) 14:813–22. doi: 10.1016/S1470-2045(13)70279-1, PMID: 23849838

[4] Bonner MR Freeman LE Hoppin JA Koutros S Sandler DP Lynch CF. Occupational exposure to pesticides and the incidence of lung Cancer in the agricultural health study. Environ Health Perspect. (2017) 125:544–51. doi: 10.1289/EHP456, PMID: 27384818 PMC5381995

[5] Molina JR Yang P Cassivi SD Schild SE Adjei AA. Non-small cell lung cancer: epidemiology, risk factors, treatment, and survivorship. Mayo Clin Proc. (2008) 83:584–94. doi: 10.1016/S0025-6196(11)60735-0, PMID: 18452692 PMC2718421

[6] Ettinger DS Wood DE Aisner DL Akerley W Bauman JR Bharat A. Non-small cell lung cancer, Version 3.2022, NCCN Clinical Practice Guidelines in Oncology. J Natl Compr Canc. Netw. (2022) 20:497–30. doi: 10.6004/jnccn.2022.0025 PMID: 35545176

[7] Bade BC Faiz SA Ha DM Tan M Barton-Burke M Cheville AL. Cancer-related fatigue in lung cancer: a research agenda: an official American Thoracic Society research statement. Am J Respir Crit Care Med. (2023) 207:e6–e28. doi: 10.1164/rccm.202210-1963ST, PMID: 36856560 PMC10870898

[8] Tur JA Bibiloni MM Sureda A Pons A. Dietary sources of omega 3 fatty acids: public health risks and benefits. Br J Nutr. (2012) 107:S23–52. doi: 10.1017/S0007114512001456 22591897

[9] Serini S Ottes Vasconcelos R Fasano E Calviello G. How plausible is the use of dietary n-3 PUFA in the adjuvant therapy of cancer? Nutr Res Rev. (2016) 29:102–25. doi: 10.1017/S0954422416000044, PMID: 27172872

[10] Deniz C Raba-Parodi C Garcia-Raimundo E Macia I Rivas F Urena A. Preoperative omega-6/omega-3 fatty acid ratio could predict postoperative outcomes in patients with surgically resected non-small-cell lung cancer. Curr Oncol. (2022) 29:7086–98. doi: 10.3390/curroncol29100556, PMID: 36290833 PMC9600895

[11] Tao X Zhou Q Rao Z. Efficacy of omega-3 polyunsaturated fatty acids in patients with lung Cancer undergoing radiotherapy and chemotherapy: a meta-analysis. Int J Clin Pract. (2022) 2022:1–11. doi: 10.1155/2022/6564466 PMC9303080 35910071

[12] Liu X Peng Y Tao R Meng L Li X. Mendelian randomization study of causal relationship between Omega-3 fatty acids and risk of lung cancer. Biomed Res Int. (2022) 2022:1–9. doi: 10.1155/2022/2786567 PMC9173898 35686230

[13] Lee KH Seong HJ Kim G Jeong GH Kim JY Park H. Consumption of fish and omega-3 fatty acids and cancer risk: an umbrella review of meta-analyses of observational studies. Adv Nutr. (2020) 11:1134–49. doi: 10.1093/advances/nmaa055, PMID: 32488249 PMC7490175

[14] Colomer R Moreno-Nogueira JM Garcia-Luna PP Garcia-Peris P Garcia-de-Lorenzo A Zarazaga A. N-3 fatty acids, cancer and cachexia: a systematic review of the literature. Br J Nutr. (2007) 97:823–31. doi: 10.1017/S000711450765795X, PMID: 17408522

[15] Candiloro F Borioli V Borsellino G Picozza M Pellini R Cereda E. Influence of different lipid emulsions on specific immune cell functions in head and neck cancer patients receiving supplemental parenteral nutrition: an exploratory analysis. Nutrition. (2021) 86:111178. doi: 10.1016/j.nut.2021.111178, PMID: 33631618

[16] Story MJ. Essential sufficiency of zinc, omega-3 polyunsaturated fatty acids, vitamin D and magnesium for prevention and treatment of COVID-19, diabetes, cardiovascular diseases, lung diseases and cancer. Biochimie. (2021) 187:94–109. doi: 10.1016/j.biochi.2021.05.013, PMID: 34082041 PMC8166046

[17] Miura K Vail A Chambers D Hopkins PM Ferguson L Grant M. Omega-3 fatty acid supplement skin cancer prophylaxis in lung transplant recipients: a randomized, controlled pilot trial. J Heart Lung Transplant. (2019) 38:59–65. doi: 10.1016/j.healun.2018.09.009, PMID: 30352778

[18] Rehman K Mohd Amin MC Yuen NP Zulfakar MH. Immunomodulatory effectiveness of fish oil and omega-3 fatty acids in human non-melanoma skin carcinoma cells. J Oleo Sci. (2016) 65:217–24. doi: 10.5650/jos.ess15256, PMID: 26876681

[19] Fearon KC Barber MD Moses AG Ahmedzai SH Taylor GS Tisdale MJ. Double-blind, placebo-controlled, randomized study of eicosapentaenoic acid diester in patients with cancer cachexia. J Clin Oncol. (2006) 24:3401–7. doi: 10.1200/JCO.2005.04.5724, PMID: 16849754

[20] Innes JK Calder PC. Marine Omega-3 (N-3) fatty acids for cardiovascular health: an update for 2020. Int J Mol Sci. (2020) 21:1362. doi: 10.3390/ijms21041362, PMID: 32085487 PMC7072971

[21] Miura K Way M Jiyad Z Marquart L Plasmeijer EI Campbell S. Omega-3 fatty acid intake and decreased risk of skin cancer in organ transplant recipients. Eur J Nutr. (2021) 60:1897–905. doi: 10.1007/s00394-020-02378-y 32909136

[22] Liu Z Ge X Chen L Sun F Ai S Kang X. The addition of omega-3 fish oil fat emulsion to parenteral nutrition reduces short-term complications after laparoscopic surgery for gastric Cancer. Nutr Cancer. (2021) 73:2469–76. doi: 10.1080/01635581.2020.1830126, PMID: 33026250

[23] Hanson S Thorpe G Winstanley L Abdelhamid AS Hooper L. Group P:Omega-3, omega-6 and total dietary polyunsaturated fat on cancer incidence: systematic review and meta-analysis of randomised trials. Br J Cancer. (2020) 122:1260–70. doi: 10.1038/s41416-020-0761-6, PMID: 32114592 PMC7156752

[24] Wei L Wu Z Chen YQ. Multi-targeted therapy of cancer by omega-3 fatty acids-an update. Cancer Lett. (2022) 526:193–204. doi: 10.1016/j.canlet.2021.11.023, PMID: 34843864

[25] Carayol M Grosclaude P Delpierre C. Prospective studies of dietary alpha-linolenic acid intake and prostate cancer risk: a meta-analysis. Cancer Causes Control. (2010) 21:347–55. doi: 10.1007/s10552-009-9465-1, PMID: 19921446

[26] Burlingame B Nishida C Uauy R Weisell R. Fats and fatty acids in human nutrition: introduction. Ann Nutr Metab. (2009) 55:5–7. doi: 10.1159/000228993 19752533

[27] Chapkin RS Kim W Lupton JR McMurray DN. Dietary docosahexaenoic and eicosapentaenoic acid: emerging mediators of inflammation. Prostaglandins Leukot Essent Fatty Acids. (2009) 81:187–91. doi: 10.1016/j.plefa.2009.05.010, PMID: 19502020 PMC2755221

[28] De Lorgeril M Salen P. New insights into the health effects of dietary saturated and omega-6 and omega-3 polyunsaturated fatty acids. BMC Med. (2012) 10:50. doi: 10.1186/1741-7015-10-50, PMID: 22613931 PMC3394202

[29] Van der Meij BS Langius JA Smit EF Spreeuwenberg MD von Blomberg BM Heijboer AC. Oral nutritional supplements containing (n-3) polyunsaturated fatty acids affect the nutritional status of patients with stage III non-small cell lung cancer during multimodality treatment. J Nutr. (2010) 140:1774–80. doi: 10.3945/jn.110.121202, PMID: 20739445

[30] Lu Y Chen RG Wei SZ Hu HG Sun F Yu CH. Effect of omega 3 fatty acids on C-reactive protein and interleukin-6 in patients with advanced nonsmall cell lung cancer. Medicine. (2018) 97:e11971. doi: 10.1097/MD.0000000000011971, PMID: 30212928 PMC6155980

[31] Pastore CA Orlandi SP Gonzalez MC. Introduction of an omega-3 enriched oral supplementation for cancer patients close to the first chemotherapy: may it be a factor for poor compliance? Nutr Cancer. (2014) 66:1285–92. doi: 10.1080/01635581.2014.956253, PMID: 25329228

[32] Murphy RA Mourtzakis M Chu QS Baracos VE Reiman T Mazurak VC. Nutritional intervention with fish oil provides a benefit over standard of care for weight and skeletal muscle mass in patients with nonsmall cell lung cancer receiving chemotherapy. Cancer. (2011) 117:1775–82. doi: 10.1002/cncr.25709, PMID: 21360698

[33] Cheng M Zhang S Ning C Huo Q. Omega-3 fatty acids supplementation improve nutritional status and inflammatory response in patients with lung cancer: a randomized clinical trial. Front Nutr. (2021) 8:686752. doi: 10.3389/fnut.2021.686752, PMID: 34395492 PMC8362886

[34] Kaya SO Akcam TI Ceylan KC Samancilar O Ozturk O Usluer O. Is preoperative protein-rich nutrition effective on postoperative outcome in non-small cell lung cancer surgery? A prospective randomized study. J Cardiothorac Surg. (2016) 11:14. doi: 10.1186/s13019-016-0407-1, PMID: 26782276 PMC4717613

[35] Murphy RA Mourtzakis M Chu QS Reiman T Mazurak VC. Skeletal muscle depletion is associated with reduced plasma (n-3) fatty acids in non-small cell lung cancer patients. J Nutr. (2010) 140:1602–6. doi: 10.3945/jn.110.123521, PMID: 20631325

[36] Lavy M Gauttier V Poirier N Barille-Nion S Blanquart C. Specialized pro-resolving mediators mitigate Cancer-related inflammation: role of tumor-associated macrophages and therapeutic opportunities. Front Immunol. (2021) 12:702785. doi: 10.3389/fimmu.2021.702785, PMID: 34276698 PMC8278519

[37] Leuzzi G Galeone C Gisabella M Duranti L Taverna F Suatoni P. Baseline C-reactive protein level predicts survival of early-stage lung cancer: evidence from a systematic review and meta-analysis. Tumori. (2016) 102:441–9. doi: 10.5301/tj.5000522, PMID: 27292573

[38] Liang B Wang S Ye YJ Yang XD Wang YL Qu J. Impact of postoperative omega-3 fatty acid-supplemented parenteral nutrition on clinical outcomes and immunomodulations in colorectal cancer patients. World J Gastroenterol. (2008) 14:2434–9. doi: 10.3748/wjg.14.2434, PMID: 18416476 PMC2705104

[39] Weiss G Meyer F Matthies B Pross M Koenig W Lippert H. Immunomodulation by perioperative administration of n-3 fatty acids. Br J Nutr. (2002) 87:S89–94. doi: 10.1079/BJN2001461 11895158

[40] Romero-Elías M Álvarez-Bustos A Cantos B Maximiano C Méndez M Méndez M. C-reactive protein is associated with physical fitness in breast cancer survivors. J Clin Med. (2023) 12:65. doi: 10.3390/jcm12010065, PMID: 36614866 PMC9821638

[41] Li Z Jin L Xia L Li X Guan Y He H. Body mass index, C-reactive protein, and pancreatic cancer: a mendelian randomization analysis to investigate causal pathways. Front Oncol. (2023) 13:1042567. doi: 10.3389/fonc.2023.1042567, PMID: 36816931 PMC9932924

[42] Miyazaki T Saji H Nakamura H Nagayasu T Okumura N Tsuchida M. The C-reactive protein to albumin ratio is a prognostic factor for stage I non-small cell lung cancer in elderly patients: JACS 1303. Surg Today. (2022) 52:1463–71. doi: 10.1007/s00595-022-02485-9 35211804

[43] Frey A Martin D D’Cruz L Fokas E Rodel C Fleischmann M. C-reactive protein to albumin ratio as prognostic marker in locally advanced non-small cell lung Cancer treated with chemoradiotherapy. Biomedicine. (2022) 10:598. doi: 10.3390/biomedicines10030598, PMID: 35327399 PMC8945805

[44] Kavyani Z Musazadeh V Fathi S Hossein Faghfouri A Dehghan P Sarmadi B. Efficacy of the omega-3 fatty acids supplementation on inflammatory biomarkers: an umbrella meta-analysis. Int Immunopharmacol. (2022) 111:109104. doi: 10.1016/j.intimp.2022.109104, PMID: 35914448

[45] Solis-Martinez O Plasa-Carvalho V Phillips-Sixtos G Trujillo-Cabrera Y Hernandez-Cuellar A Queipo-Garcia GE. Effect of eicosapentaenoic acid on body composition and inflammation markers in patients with head and neck squamous cell Cancer from a public Hospital in Mexico. Nutr Cancer. (2018) 70:663–70. doi: 10.1080/01635581.2018.1460678, PMID: 29697274

